# Retraction: Plumbagin Ameliorates Collagen-Induced Arthritis by Regulating Treg/Th17 Cell Imbalances and Suppressing Osteoclastogenesis

**DOI:** 10.3389/fimmu.2020.00112

**Published:** 2020-01-23

**Authors:** Frontiers Editorial Office

The journal retracts the January 8, 2019 article cited above.

Following publication, concerns regarding the integrity of the western blot images were brought to the attention of the Publisher. After an investigation by the Editorial Office and Chief Editors of the journal, image duplication and manipulation was revealed in Figure 6. The authors have failed to provide a satisfactory explanation following several requests. The authors do not agree to the retraction or the notice.

The retraction of the article was approved by the Chief Editors of Frontiers in Immunology and the Editor-in-Chief of Frontiers.

